# Modular cell-based platform for high throughput identification of compounds that inhibit a viral interferon antagonist of choice

**DOI:** 10.1016/j.antiviral.2017.10.012

**Published:** 2018-02

**Authors:** Andri Vasou, Christina Paulus, Janina Narloch, Zoe O. Gage, Marie-Anne Rameix-Welti, Jean-François Eléouët, Michael Nevels, Richard E. Randall, Catherine S. Adamson

**Affiliations:** a School of Biology, Biomedical Sciences Research Complex, University of St Andrews, St Andrews, KY16 9ST, United Kingdom; b UMR INSERM U1173 2I, UFR des Sciences de la Santé Simone Veil—UVSQ, 78180, Montigny-Le-Bretonneux, France; c AP-HP, Laboratoire de Microbiologie, Hôpital Ambroise Paré, 92104, Boulogne-Billancourt, France; d Unité de Virologie et Immunologie Moléculaires (UR892), INRA, Université Paris-Saclay, 78352, Jouy-en-Josas, France

**Keywords:** Viral interferon (IFN) antagonists, Antivirals, Human respiratory syncytial virus (RSV), Human cytomegalovirus (CMV), High-throughput screening (HTS), Signal transducer and activator of transcription 2 (STAT2)

## Abstract

Viral interferon (IFN) antagonists are a diverse class of viral proteins that counteract the host IFN response, which is important for controlling viral infections. Viral IFN antagonists are often multifunctional proteins that perform vital roles in virus replication beyond IFN antagonism. The critical importance of viral IFN antagonists is highlighted by the fact that almost all viruses encode one of these proteins. Inhibition of viral IFN antagonists has the potential to exert pleiotropic antiviral effects and thus this important protein class represents a diverse plethora of novel therapeutic targets. To exploit this, we have successfully developed and executed a novel modular cell-based platform that facilitates the safe and rapid screening for inhibitors of a viral IFN antagonist of choice. The platform is based on two reporter cell-lines that provide a simple method to detect activation of IFN induction or signaling via an eGFP gene placed under the control of the IFNβ or an ISRE-containing promoter, respectively. Expression of a target IFN antagonist in the appropriate reporter cell-line will block the IFN response and hence eGFP expression. We hypothesized that addition of a compound that inhibits IFN antagonist function will release the block imposed on the IFN response and hence restore eGFP expression, providing a measurable parameter for high throughput screening (HTS). We demonstrate assay proof-of-concept by (i) exploiting hepatitis C virus (HCV) protease inhibitors to inhibit NS3-4A's capacity to block IFN induction and (ii) successfully executing two HTS targeting viral IFN antagonists that block IFN signaling; NS2 and IE1 from human respiratory syncytial virus (RSV) and cytomegalovirus (CMV) respectively, two clinically important viruses for which vaccine development has thus far been unsuccessful and new antivirals are required. Both screens performed robustly and Z′ Factor scores of >0.6 were achieved. We identified (i) four hit compounds that specifically inhibit RSV NS2's ability to block IFN signaling by mediating STAT2 degradation and exhibit modest antiviral activity and (ii) two hit compounds that interfere with IE1 transcription and significantly impair CMV replication. Overall, we demonstrate assay proof-of-concept as we target viral IFN antagonists from unrelated viruses and demonstrate its suitability for HTS.

## Highlights

• Viral IFN antagonists represent a plethora of novel therapeutic targets not specifically targeted by current antivirals.
• We developed a novel modular cell-based screening platform that potentially targets any viral IFN antagonist of choice.
• The assay is based on eGFP reporter gene expression at the end-point of activated IFN induction and signaling pathways.
• We demonstrate assay proof-of-concept via HCV protease inhibitors, which block NS3-4A's capacity to block IFN induction.
• We successfully execute two high-throughput screens targeting IFN antagonists NS2 and IE1 from RSV and CMV, respectively.

## 1 Introduction

Viral interferon (IFN) antagonists are a vital protein class not specifically targeted by clinically approved antivirals (De Clercq and Li, 2016). These diverse viral proteins counteract the host IFN system, a powerful innate immune response important for controlling viral infections. Upon virus infection, IFN expression is triggered. Secreted IFN stimulates signaling to activate expression of IFN-stimulated genes (ISGs), which elicit an antiviral state (Hoffmann et al., 2015, Randall and Goodbourn, 2008).

Viruses have evolved a wide variety of strategies to circumvent the IFN response (Beachboard and Horner, 2016). The critical importance of viral IFN antagonists is highlighted by the fact that almost all viruses encode at least one antagonist (Versteeg and Garcia-Sastre, 2010). Genetic studies have demonstrated the importance of viral IFN antagonists in virus replication, virulence and pathogenesis (Fleming, 2016). Disabling viral IFN antagonist function impedes a virus' ability to counteract the IFN response, predisposing infection in favor of the host and consequently virus clearance. In addition, viral IFN antagonists are often multifunctional proteins that perform vital roles in virus replication beyond IFN antagonism (Fehling et al., 2012, Hale et al., 2008). Therefore, inhibition of viral IFN antagonists has the potential to exert pleiotropic antiviral effects.

To exploit the abundant array of viral IFN antagonists as potential drug targets our objective was development of a novel modular cell-based platform that facilitates safe and rapid screening for inhibitors against any viral IFN antagonist of choice. Towards this aim we previously generated two reporter cell-lines, A549/pr(IFNβ).GFP and A549/pr(ISRE).GFP, that provide a simple method to detect activation of IFN induction or signaling via an eGFP gene under the control of the IFNβ or an ISRE-containing promoter, respectively (Chen et al., 2010, Stewart et al., 2014) and demonstrated their suitability for high-throughput screening (HTS) (Gage et al., 2016). Here we utilize these validated reporter cell-lines as a platform to target viral IFN antagonists. We have shown that viral IFN antagonist expression in the A549/pr(IFNβ).GFP reporter cell-line blocks the IFN response and hence eGFP expression (Chen et al., 2010). We hypothesized that addition of a compound that inhibits IFN antagonist function will release the imposed block and hence restore eGFP expression, providing a measurable parameter for HTS.

For initial proof-of-concept we exploit hepatitis C virus (HCV) protease inhibitors (PIs); antivirals that inhibit NS3-4A (De Clercq and Li, 2016), an HCV protein with IFN antagonist function (Xu and Zhong, 2016). PI inhibition of NS3-4A prevents cleavage of the HCV polyprotein and critical MAVS/TRIF components of the IFN induction pathway (Kalkeri et al., 2013). For HTS we target IFN antagonists from two clinically important human viruses, respiratory syncytial virus (RSV) and cytomegalovirus (CMV), for which vaccine development has thus far been unsuccessful and new antivirals are required (Griffiths et al., 2015, Griffiths et al., 2017). We target the RSV IFN antagonist non-structural protein 2 (NS2), which blocks IFN signaling via proteasomal degradation of signal transducer and activator of transcription 2 (STAT2) (Barik, 2013, Lo et al., 2005, Ramaswamy et al., 2006). NS2 also plays an important role in RSV pathogenicity independent of IFN antagonism (Liesman et al., 2014, Yang et al., 2015). We further target the CMV IFN antagonist immediate-early 1 protein (IE1), which counteracts IFN signaling by binding directly to STAT2 thereby preventing the transcription factor complex ISGF3 from binding ISRE elements in the promoters of ISGs (Paulus et al., 2006). IE1 also plays a crucial role in the initiation of lytic CMV infection as IE1, along with IE2, stimulate the transcription of early genes required to replicate viral DNA (Stinski and Meier, 2007).

## 2 Materials and methods

### 2.1 Cell-lines

The A549 cell-line (ECACC) and derivatives (Appendix Table A.1) were maintained in DMEM with 10% fetal bovine serum (FBS) (DMEM-10). A549 reporter cell-line derivatives expressing HCV NS3-4A, RSV NS1/NS2 or PIV5 V were generated using a self-inactivating lentiviral constitutive expression system (Demaison et al., 2002). A549 reporter cell-line derivatives expressing CMV IE1 and corresponding negative controls (luciferase (Luc) and IE1dl410-420 (Harwardt et al., 2016)) were generated using the Lenti-X Tet-One inducible expression system (Clontech). Lentiviruses were generated in 293T cells (ECACC), used to transduce the appropriate reporter cell-line and maintained under antibiotic selection. A Hep2 cell-line derivative constitutively expressing PIV2 V (Precious et al., 2005) or BVDV Npro (Hilton et al., 2006), MRC-5 cells (ATCC), Vero cells (ECACC) and STAT2-deficient primary skin fibroblasts (Hambleton et al., 2013) were maintained in DMEM-10.

### 2.2 Cell-based IFN induction and signaling reporter assays

The A549/pr(IFNβ).GFP and A549/pr(ISRE).GFP reporter assays have been described (Gage et al., 2016, Stewart et al., 2014). Briefly, 24 h post cell seeding (9 × 10^4^ cells/cm^2^) the A549/pr(IFNβ).GFP cell-line is activated by infection with a PIV5VΔC stock rich in defective interfering (DI) particles (He et al., 2002) or a UV-inactivated pp65-deficient CMV stock (Buscher et al., 2015) and the A549/pr(ISRE).GFP cell-line is activated with 10^4^ U/ml purified IFNα (Roferon, NHS). A549 reporter cell-line derivatives expressing inducible CMV IE1, Luc or IE1dl410-420 were seeded in the presence or absence of doxycycline (1 μg/ml). When appropriate, cells were treated with compound 2 h prior to activation of IFN induction or signaling and assay output was measured in raw fluorescent units (RFU) using an Infinite-M200-Pro plate reader (Tecan).

### 2.3 Compound library, inhibitors and cell viability assays

The compound library used for HTS, termed StA-HTS-Library, was a kind gift of Professor Westwood, University of St Andrews. The library (n = 16,000 compounds at 10 mM in DMSO) consists of the Maybridge HitFinder™ Collection (n = 14,400 compounds, ThermoFisher Scientific; selected by Maybridge to represent drug-like diversity of the Maybridge Screening Collection) supplemented with 1600 compounds that have been synthesized in-house by Professor Westwood. Hit compounds StA-NS2-1 (ethyl2-(1H-indol-3 ylmethylidene)hydrazine-1-carboxylate), StA-NS2-2 (N-(1H-indazol-3-yl)-3-methoxybenzamide), StA-NS2-3 (1H-indol-3-yl)butan-2-one), StA-NS2-4 (methyl 2-aminothiophene-3-carboxylate), StA-IE1-1 (7-(4-fluorophenyl)-6a,7-dihydro-6H-chromeno[3,4-c][1,5]benzothiazepine), StA-IE1-2 (1-(3-nitrophenyl)-2-(pyrido[3,2-d][1,3]thiazol-2-ylthio)ethan-1-one), and StA-IE1-3 (1-(3,5-dichloro-4-pyridinyl)-4-piperidinecarboxamide) where repurchased from Maybridge (ThermoFisher Scientific) and stored as 20 mM stocks in DMSO. Compound purity was determined by the manufacturer and ranged from 90 to 97%. HCV PI Danoprevir (Selleckchem), RSV RNA polymerase inhibitor ASL-8112 (MedChemExpress) and CMV DNA polymerase inhibitor ganciclovir (GCV) (Calbiochem) were stored as 20 mM stock in DMSO. Commercial cell-viability assays (AlamarBlue, PrestoBlue (ThermoFisher Scientific) or CellTiter 96 AQueous One Solution (Promega)) were performed according to manufacturer instructions. Crystal violet staining determined cell density by measuring absorbance at 570 nm (Infinite-M200-Pro plate reader, Tecan).

### 2.4 HTS and dose-response assays

Single-point HTS was performed using the A549/pr(ISRE).GFP-RSV-NS2 and A549/pr(ISRE).GFP-CMV-IE1 cell-lines and the StA-HTS-Library. The A549/pr(ISRE).GFP reporter assay was used with the following variations. Cells were seeded (9 × 10^4^ cells/cm^2^) in 384-well plates and 11 μM of compound was added using a MiniTrak liquid handler (PerkinElmer) 2 h prior to IFNα activation. HTS screening data output was analyzed as fold-increase in eGFP expression calculated as:

(A.1) Fold increase eGFP = μ (RFU_Test_)/μ (RFU_Uninduced_)

HTS quality control criteria for the acceptance of an assay plate were as follows: signal-to-background (S/B) ratio ≥2-fold, coefficient of variation (CV) < 8% ((σ/μ)*100) and Z′ Factor ≥0.5 (Eq. (A2)) (Gage et al., 2016). Each appropriate statistical parameter was determined for each assay plate and the overall screen.

(A.2) Z′ Factor = 1−[(3 (σ_Uninduced_ + σ_Induced_))/| μ_Uninduced_−μ_Induced_ |]

Dose response curves were performed via nine-point two-fold serial dilution (50–0.1 μM). For determination of potency, a four-parameter logical fit of the following form was used:

(A.3) Y = B + [(T−B)/(1 + (EC_50_)/X)^h^]

B = baseline response (bottom asymptote), T = maximum response (top asymptote), h = slope (Hill coefficient) and EC_50_ = compound concentration 50% between baseline and maximum.

### 2.5 Protein analyses

Preparation of whole cell protein, SDS-PAGE, immunoblotting, indirect immunofluorescence microscopy and co-immunoprecipitation analyses were performed according to previously published protocols (Gage et al., 2016, Harwardt et al., 2016, Krauss et al., 2009, Nevels et al., 2004). Appendix Table A.2 provides details of primary antibodies. HRP-conjugated secondary antibodies were used for visualization via enhanced chemiluminesence and IRDye 800CW- or IRDye 680RD-conjugated secondary antibodies were used for visualization and quantification via an Odyssey CLx near-infrared scanner (Li-Cor).

### 2.6 Virological assays

rRSV-WT, rRSV-GFP (Rameix-Welti et al., 2014) and PIV5mCherry (kind gift of Dr He, University of Georgia, USA) were propagated in Hep2-BVDV/Npro cells (Hilton et al., 2006) and stock titer determined. Virus infections were performed at the indicated MOI and, where appropriate, treated at the indicated time and concentration with StA-NS2-2, DMSO and/or 10^4^ U/ml IFNα. rRSV-GFP and PIV5mCherry virus replication was monitored overtime by (i) eGFP or mCherry expression respectively, using an Infinite-M200-Pro plate reader or (ii) determining rRSV-GFP infectious titer (PFU/ml) in supernatant harvested at 24, 48, 72 and 96 h post infection. Infectious titer was determined via plaque assay using Hep2-BVDV-Npro cells and a 1% methyl cellulose (Sigma-Aldrich) overlay in DMEM supplemented with 2% FBS. Plaques were visualized after 4 days using a BioRad ChemiDoc MP imaging system and an XcitaBlue conversion screen. CMV gTBwt, an eGFP expressing version of the TB40E strain (Harwardt et al., 2016, Sinzger et al., 2008, Umashankar et al., 2011), was reconstituted and virus stocks were produced on electroporation of MRC-5 cells with BAC DNA. Stock titer was determined and IE1 mRNA levels measured by RT-qPCR with primers 471 and 472 as described in Harwardt et al. (2016) with the following modifications. The 2 × concentrated Brilliant III Ultra-Fast SYBR Green Low ROX QPCR Master Mix and Mx3005P QPCR System (Agilent Technologies) were used. The relative amount of viral DNA in culture supernatants was determined using procedures previously described (Harwardt et al., 2016). For CMV plaque reduction assays human fibroblasts were pretreated with compound or solvent 2 h before inoculation with 50 PFU of gTBwt. Infection was performed for 2 h in the presence of solvent or compound. Post-infection cells were overlaid with 1% methyl cellulose in DMEM supplemented with 2% FBS and the appropriate concentration of solvent or compound. Cultures were incubated for 7 days (MRC-5 cells) or 11 days (STAT2-deficient dermal fibroblasts) before plaques were counted using an EVOS FL Cell Imaging System with Plan achromat 4 × Objective and GFP Light Cube. Foci of three or more fluorescent cells were counted as plaques. All plaque assays were performed in triplicate.

## 3 Results and discussion

### 3.1 Assay proof-of-concept using HCV PIs

**Fig. 1:**
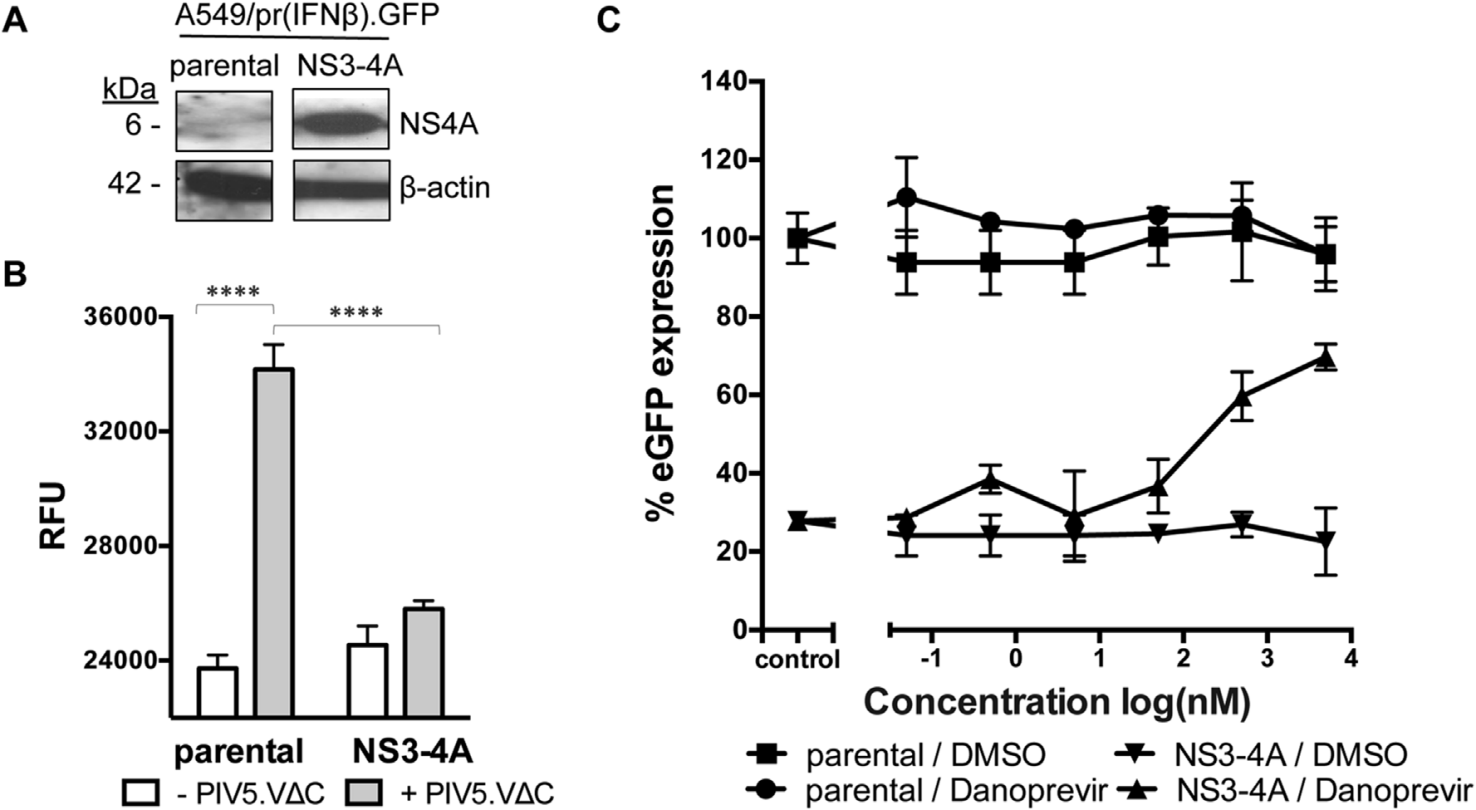
**Restoration of eGFP expression in activated A549/pr(IFNβ).GFP-NS3-4A reporter cells by the HCV PI Danoprevir. (A)** Detection of NS4A and β-actin in A549/pr(IFNβ).GFP or A549/pr(IFNβ).GFP-NS3-4A reporter cells. Cells were lysed and subjected to SDS-PAGE/Western blot, followed by detection with anti-V5 or anti-actin antibody and HRP-conjugated secondary antibody. Bands were visualized by chemiluminescence. **(B)** Effect of NS3-4A on eGFP reporter gene expression upon activation of the IFN induction pathway. A549/pr(IFNβ).GFP or A549/pr(IFNβ).GFP-NS3-4A reporter cells were infected with PIV5.VΔC to activate the IFN induction pathway. Sixteen hours post-infection eGFP expression was measured as raw fluorescent units (RFU). Statistical significance was assessed using a two-way ANOVA test: ****, p < 0.0001. Data shown represents mean values (n = 6 replicates; error bars = SD) and is representative of at least 3 independent experiments. **(C)** Effect of HCV PI Danoprevir on restoration of eGFP expression upon activation of the IFN induction pathway. A549/pr(IFNβ).GFP or A549/pr(IFNβ).GFP-NS3-4A reporter cells were treated with Danoprevir using a 6-point dose response curve (10-fold dilution series 0.05 nM-5 μM) or equivalent volume of DMSO (control). Two hours post-compound treatment cells were infected with PIV5VΔC. Sixteen hours post-infection eGFP expression was measured and % eGFP calculated relative to non-treated/PIV5.VΔC-infected A549/pr(IFN-β)GFP reporters cells, which represent 100%. Data shown represents mean values (n = 3 replicates; error bars = SD) and is representative of at least 3 independent experiments.

We hypothesized that our validated A549/pr(IFNβ).GFP and A549/pr(ISRE).GFP reporter assays (Gage et al., 2016, Stewart et al., 2014) may be used as a platform to target viral IFN antagonists to discover novel antiviral compounds. To test this we generated a A549/pr(IFNβ).GFP cell-line derivative constitutively expressing HCV genotype 1b NS3-4A (A549/pr(IFNβ).GFP-NS3-4A) (Fig. 1A) and exploited HCV PIs inhibiting NS3-4A's capacity to block IFN induction (Kalkeri et al., 2013). As expected, PIV5VΔC infection of the A549/pr(IFNβ).GFP cell-line activated the IFN induction pathway and hence eGFP expression (Fig. 1B). In contrast, eGFP expression in PIV5VΔC-infected A549/pr(IFNβ).GFP-NS3-4A cells was almost completely blocked, indicating that NS3-4A is functional in the reporter cell-line (Fig. 1B). Addition of HCV PI Danoprevir (Deutsch and Papatheodoridis, 2010) to PIV5VΔC-infected A549/pr(IFNβ).GFP-NS3-4A cells resulted in a dose-dependent restoration of eGFP expression (Fig. 1C). These results provide proof-of-concept that addition of a compound inhibiting IFN antagonist function can release the block imposed on the IFN response pathway via restoration of eGFP expression, providing a measurable parameter for HTS.

### 3.2 HTS for inhibitors of RSV NS2

**Fig. 2:**
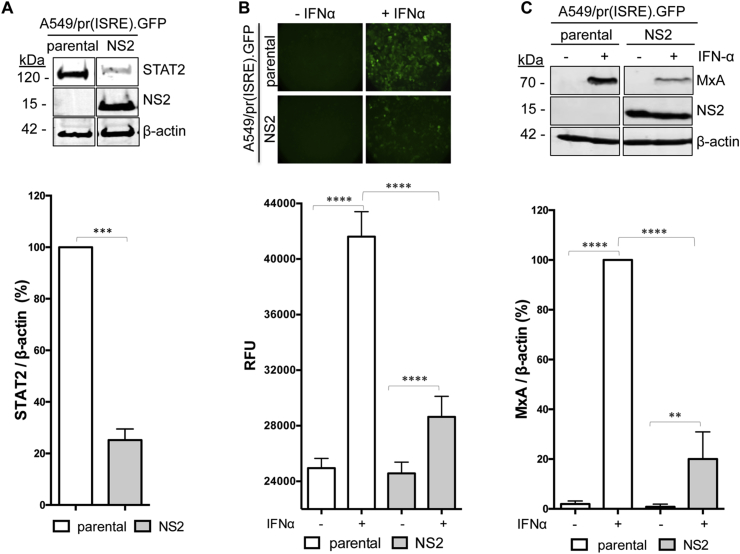
**Characterization of the A549/pr(ISRE).GFP-NS2 reporter cell-line. (A)** Detection of NS2, STAT2 and β-actin in A549/pr(IFNβ).GFP or A549/pr(ISRE).GFP-NS2 reporter cells. Cells were lysed and subjected to SDS-PAGE/Western blot, followed by detection with anti-myc, anti-STAT2 and anti-β-actin antibody and IRDye680-conjugated secondary antibody. Bands were visualized using an Odyssey near-infrared scanner and STAT2 levels quantified as % STAT2 relative to β-actin. Statistical significance was assessed using a two-tailed paired T-test; ***, p < 0.0005. Data shown represents mean values from four independent experiments; error bar = SD. (**B**) Effect of NS2 on eGFP reporter gene expression upon activation of the IFN signaling pathway. A549/pr(ISRE).GFP or A549/pr(ISRE).GFP-NS2 reporter cells were incubated with IFNα to activate the IFN signaling pathway. Forty-eight hours post-IFNα treatment, eGFP expression was visualized and measured as raw fluorescent units (RFU). Data shown represents three independent experiments each with 12 replicates; error bars = SD. Statistical significance was assessed using a two-way ANOVA with Tukey's multiple comparisons test; ****, p < 0.0001. (**C**) Effect of NS2 on ISG MxA expression upon activation of the IFN signaling pathway. A549/pr(ISRE).GFP or A549/pr(ISRE).GFP-NS2 reporter cells were incubated with IFNα. Sixteen hours post-IFNα treatment, cell lysates were subjected to SDS-PAGE/Western blot followed by detection with anti-MxA, anti-myc or anti-actin antibody and IRDye800-conjugated secondary antibody. Bands were visualized using an Odyssey near-infrared scanner and MxA levels quantified as % MxA relative to β-actin. Statistical significance was assessed using a two-way ANOVA with Tukey's multiple comparisons test; ****, p < 0.0001, **, p < 0015. Data shown represents mean values from four independent experiments; error bar = SD.

To target RSV NS2 we generated an A549/pr(ISRE).GFP derivative that constitutively expresses NS2 (Fig. 2A). STAT2 levels are significantly reduced in the A549/pr(ISRE).GFP-NS2 cell-line (Fig. 2A), confirming that NS2 antagonizes IFN signaling via STAT2 proteasomal degradation. As expected, IFN signaling was activated in IFN-treated A549/pr(ISRE).GFP cells and resulted in eGFP expression (Fig. 2B). In contrast, eGFP expression in IFN-treated A549/pr(ISRE).GFP-NS2 cells was significantly reduced, indicating that NS2 is functional in the reporter cell-line (Fig. 2B). Levels of the ISG MxA were also significantly reduced in IFN-treated A549/pr(ISRE).GFP-NS2 cells (Fig. 2C), confirming NS2 functionality independent of the eGFP reporter gene. Thus the A549/pr(ISRE).GFP-NS2 reporter cell-line is suitable for an HTS campaign to target NS2.

**Fig. 3:**
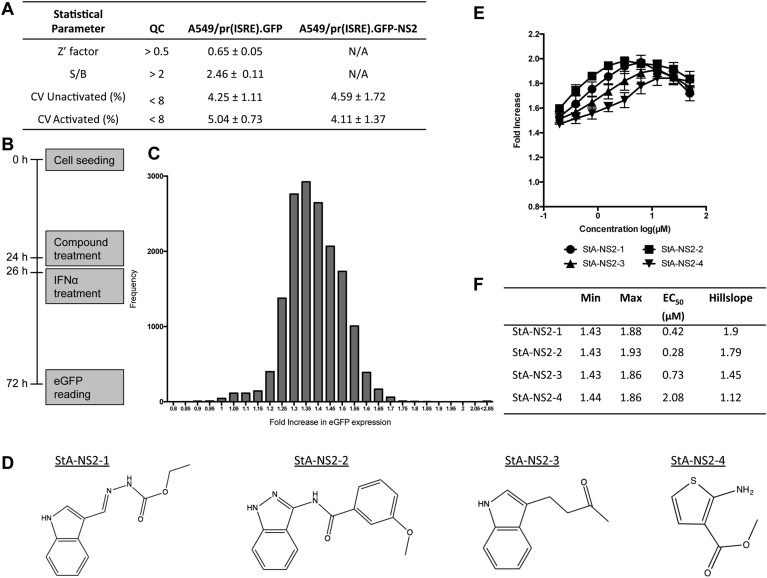
**Single-point high-throughput screen to identify compounds that inhibit RSV NS2-mediated antagonism of the IFN signaling pathway. (A)** Screen statistics for the A549/pr(ISRE).GFP and A549/pr(ISRE).GFP-NS2 reporter assays compared with preset quality control (QC) standards. S/B, signal-to-background ratio; CV, coefficient of variation. (**B**) HTS assay procedure and timing of treatments. (**C**) A single point HTS screen was performed using the A549/pr(ISRE).GFP-NS2 reporter cell-line and StA-HTS-Library (n = 16,000 compounds). Screen output, represented as fold increase in eGFP expression and plotted as a frequency distribution of all compounds tested. (**D**) Repurchased hit compound chemical structures. (**E**) Hit compound retesting using the A549/pr(ISRE).GFP-NS2 reporter assay and a 10-point dose-response curve (two-fold serial dilution series 50–0.1 μM). Data shown represents mean values (n = 6 replicates; error bars = SD) and is representative of at least 3 independent experiments. (**F**) Dose-response curve-generated EC_50_ (μM), maximum, minimum and Hill slope values using Prism 6 Software (GraphPad Software).

A single-point primary HTS against the A549/pr(ISRE).GFP-NS2 cell-line was performed using the StA-HTS-Library. We first confirmed that the A549/pr(ISRE).GFP reporter assay is suitable for HTS via Z′ Factor, S/B ratio and CV analysis (Fig. 3A and (Gage et al., 2016)). Following this analysis, the A549/pr(ISRE).GFP-NS2 cell-line was used to conduct the HTS (Fig. 3B). Due to a lack of an existing positive control for NS2 inhibition, each well contained IFN-treated A549/pr(ISRE).GFP-NS2 cells and a single test compound. The lack of a positive control of NS2 inhibition also meant that statistical validation of the A549/pr(ISRE).GFP-NS2 cell-line via S/B ratio and Z′ Factor is not possible as NS2 expression blocks eGFP expression and hence the signal window required to calculate these parameters. However, CV analysis remained valid and was within the HTS quality control threshold (Fig. 3A), indicating that NS2 expression does not alter assay variability. A normal distribution of fold-increase in eGFP expression was observed, indicating that the screen performed as expected (Fig. 3C). A hit was designated as having restored eGFP expression levels ≥1.7-fold above the non-activated A549/pr(ISRE).GFP-NS2 cell-line level and ≥3 SDs above the test well average for the plate. These criteria resulted in 28 putative hit compounds and a primary screen hit rate of 0.18%.

These 28 compounds were cherry-picked from the StA-HTS-Library and re-tested by dose response screening in (i) the A549/pr(ISRE).GFP-NS2 reporter assay to confirm eGFP restoration and (ii) cell-free media to identify auto-fluorescent compounds. Twenty compounds were eliminated due to auto-fluorescence or lack of activity upon re-testing. The remaining 8 compounds exhibited dose-dependent eGFP restoration without auto-fluoresence and the best four compounds (Fig. 3D), named StA-NS2-1, -2, -3 and -4, repurchased. Repurchased compounds exhibited dose-dependent eGFP restoration in IFN-treated A549/pr(ISRE).GFP-NS2 cells (Fig. 3E) with EC_50_ values in the μM range (Fig. 3F).

To eliminate the possibility that the four hit compounds were false positives we demonstrated that the repurchased compounds did not: (i) exhibit auto-fluorescence, (ii) cause non-specific up-regulation of eGFP, (iii) disrupt NS2 expression or proteolytic processing, (iv) affect cell viability (Appendix Fig. B.1). Overall, our data suggest that compounds StA-NS2-1, -2, -3 and -4 can be considered confirmed hits.

### 3.3 Hit compounds specifically inhibit RSV NS2 function and exhibit antiviral activity

**Fig. 4:**
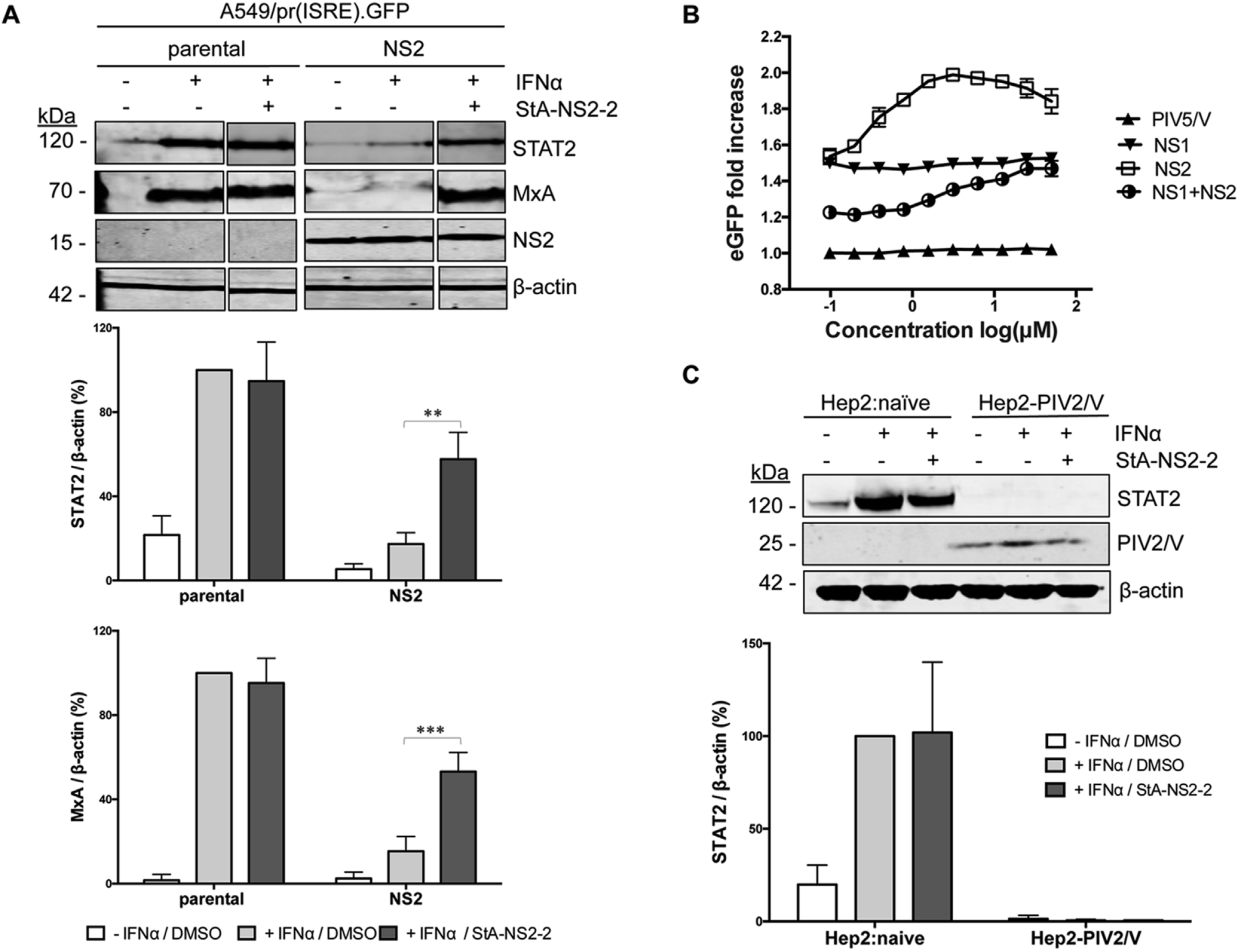
**Specificity of hit compound StA-NS2-2. (A)** Effect of StA-NS2-2 on MxA and STAT2 levels. A549/pr(ISRE).GFP and A549/pr(ISRE).GFP-NS2 cells were treated with StA-NS2-2 or equivalent volume of DMSO. Two hours post-compound treatment, IFNα was used to activate the IFN signaling pathway. Sixteen hours post-IFNα, cells were lysed, subjected to SDS-PAGE/Western blot, followed by detection with anti-STAT2, anti-MxA, anti-myc or anti-actin antibody and IRDye800-conjugated secondary antibody. Bands were visualized using an Odyssey near-infrared scanner, followed by quantification of % STAT2 and % MxA relative to β-actin. Data shown represents mean values from 3 independent experiments; error bars = SD. Statistical significance was assessed using two-way ANOVA with Turkey's multiple comparison test to compare effect of StA-NS2-2 on STAT2 or MxA levels in αIFN-treated A549/(ISRE).GFP-NS2 cells; ***, p < 0.0005, **, p < 0.005. (**B**) StA-NS2-2 activity is specific to RSV NS2 and not unrelated viral IFN antagonists RSV NS1 or PIV5 V. A549/pr(ISRE).GFP derivatives expressing RSV NS2, RSV NS1, RSV NS1+NS2 or PIV5 V were treated with StA-NS2-2 using 10-point dose-response curve (two-fold serial dilution series 50–0.1 μM). Two hours post-compound treatment, IFNα treatment activated the IFN signaling pathway. Forty-eight hours post-IFNα treatment, eGFP expression was measured and fold-increase calculated. Data shown represents mean values (n = 4 replicates; error bars = SD) and is representative of at least 3 independent experiments. (**C**) StA-NS2-2 activity is specific to RSV NS2 and not PIV2 V protein. Hep2 and Hep2-PIV2/V cell-lines were treated with StA-NS2-2 or equivalent volume of DMSO. Two hours post-compound treatment, IFNα was used to activate the IFN signaling pathway. Sixteen hours post-IFNα treatment, cells were lysed, subjected to SDS-PAGE/Western blot, followed by detection with anti-STAT2, anti-V5 or anti-actin antibody and IRDye800-conjugated secondary antibody. Bands were visualized using an Odyssey near-infrared scanner, followed by quantification of % STAT2 relative to β-actin. Data shown represents mean values from 3 independent experiments; error bars = SD.

As compounds StA-NS2-1, -2 and -3 are structurally related (Fig. 3D) only StA-NS2-2 was taken forward for further study. StA-NS2-2 raised MxA and STAT2 levels in IFN-treated A549/pr(ISRE).GFP-NS2 cells (Fig. 4A), suggesting that StA-NS2-2 partially restores the IFN signaling pathway by inhibiting NS2 function.

StA-NS2-2 specificity against RSV NS2 was investigated using A549/pr(ISRE).GFP cell-line derivatives expressing unrelated viral IFN antagonists that block IFN signaling. Derivatives used constitutively express (i) PIV5 IFN antagonist V (Didcock et al., 1999), (ii) RSV's other IFN antagonist non-structural protein 1 (NS1), which imposes a moderate IFN signaling block when expressed independently from NS2 but not via STAT2 degradation (Lo et al., 2005, Ramaswamy et al., 2006, Swedan et al., 2009) and (iii) a double reporter cell-line that expresses RSV NS1 and NS2. Dose-dependent eGFP restoration was only observed when NS2 was present, either independently or in combination with NS1 (Fig. 4B). The inability of StA-NS2-2 to reverse the block imposed by PIV5 V, which mediates STAT1 degradation (Didcock et al., 1999), suggests that the compound is not a general inhibitor of proteasomal machinery. To further verify this we show that StA-NS2-2 does not reverse the IFN signaling block imposed by PIV2 V, which like NS2 mediates the block via STAT2 proteasomal degradation (Andrejeva et al., 2002) (Fig. 4C). Our data suggest that StA-NS2-2 specifically inhibits RSV NS2 activity.

**Fig. 5:**
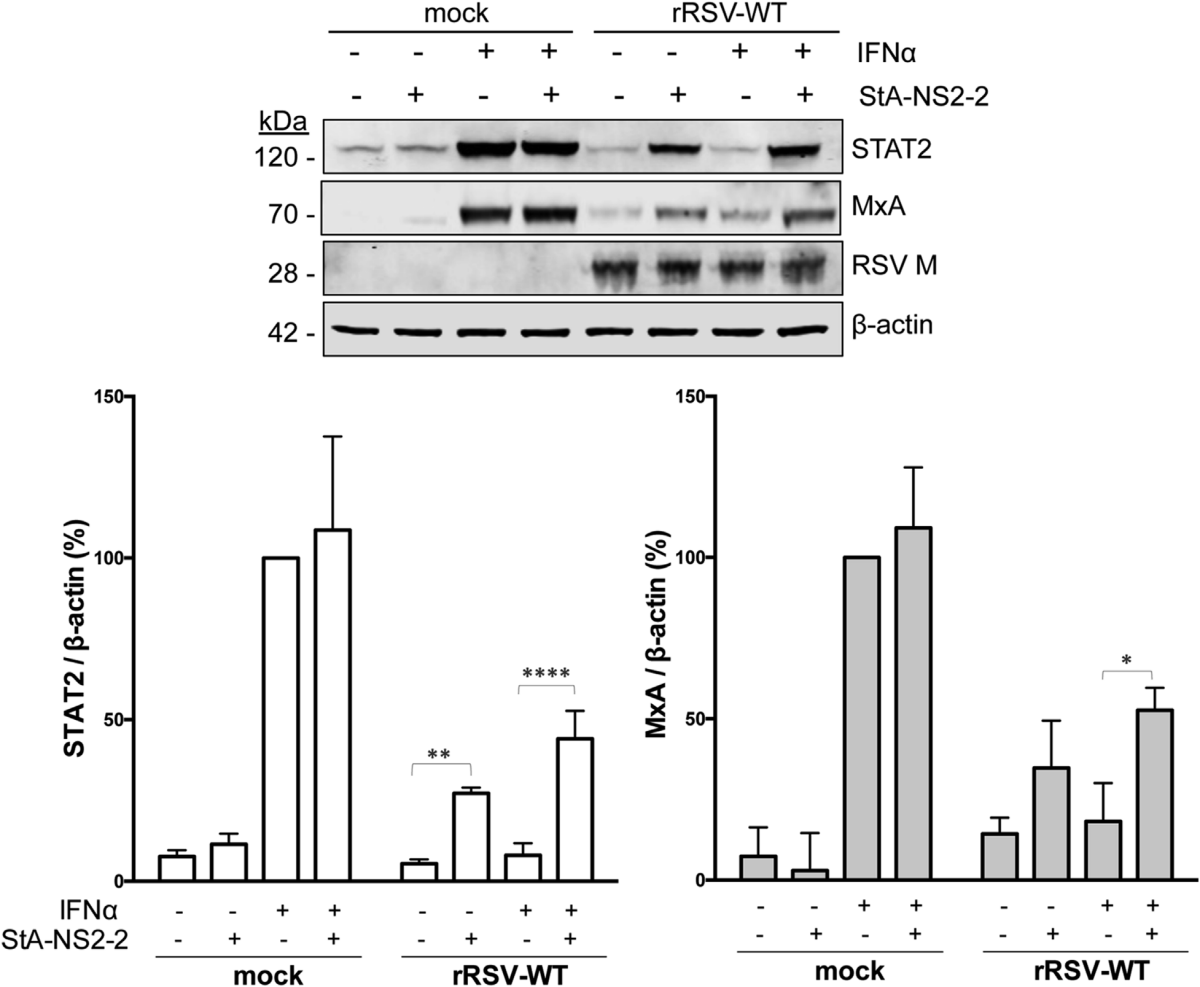
**Effect of StA-NS2-2 in the context of rRSV-WT infection.** Effect of StA-NS2-2 on MxA and STAT2 levels following infection of A549 cells with rRSV-WT. Infected cells were treated with StA-NS2-2 or the equivalent volume of DMSO. Six hours post-compound treatment, IFNα was used to activate the IFN signaling pathway. Twelve-hours post-IFNα treatment, cells were lysed, subjected to SDS-PAGE/Western blot, followed by detection with anti-STAT2, anti-MxA, anti-RSV-M or anti-actin antibody and IRDye680-or IRDye800-conjugated secondary antibody. Bands were visualized using an Odyssey near-infrared scanner, followed by quantification of % STAT2 and MxA levels relative to β-actin. Data shown represents mean values from 3 independent experiments; error bars = SD. Statistical significance was assessed using a one-way ANOVA with Tukey's multiple comparison test to compare effect of StA-NS2-2 on STAT2 and MxA levels in rRSV-WT infected cells; *, p < 0.05, **, p < 0.005, ****, p < 0.0001.

**Fig. 6:**
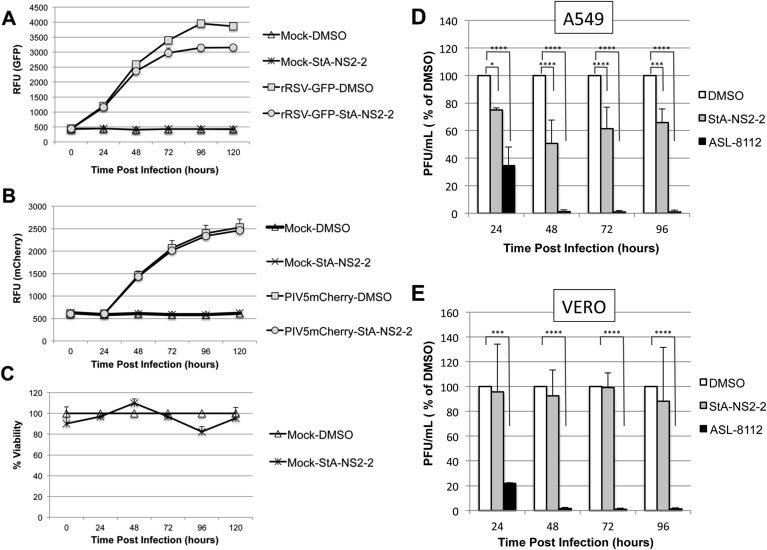
**Effect of StA-NS2-2 on replication of rRSV-GFP and PIV5mCherry.** A549 cells were treated with StA-NS2-2 (5 μM) or the equivalent volume of DMSO 24 h prior to infection with (**A**) rRSV-GFP or (**B**) PIV5mCherry at an MOI of 2.5 for 1 h at 37 °C. Mock-infected cells were subjected to the same procedure but incubated with media instead of virus stock. Following infection cells were maintained in DMEM containing 1% FBS and StA-NS2-2 (5 μM) or the equivalent volume of DMSO. Fluorescence (GFP or mCherry) was monitored as RFU every 24 h post-infection. (**C**) In parallel, mock-infected A549 cells were treated as above and % cell viability monitored using PrestoBlue cell viability reagent at each time point. DMSO-treated cells were set at 100% viability. Data shown represents mean values (n = 4 replicates; error bars = SD) and is representative of at least 3 independent experiments. (**D and E**) A549 or Vero cells were treated with StA-NS2-2 (5 μM) or the equivalent volume of DMSO 24 h prior to infection with rRSV-GFP (MOI 1). Following infection cells were maintained in DMEM containing 1% FBS and StA-NS2-2 (5 μM) or the equivalent volume of DMSO. At 24, 48, 72 and 96 h post infection supernatant was collected and stored at −80 °C. Infectious titer was determined by plaque assay using HEP2-BVDV-Npro cells. Each plaque assay was performed in triplicate and average titer (PFU/mL) determined. Data is presented as % of DMSO, which is set at 100% and represents the average of 3 independent experiments (n = 3; error bars = SD). Statistical significance was assessed using two-way ANOVA with Dunnett's multiple comparison test to compare effect of test compounds compared to DMSO control; *, p < 0.05, ***, p < 0.001, ****, p < 0.0001.

Next, we determined StA-NS2-2 activity in the context of rRSV-WT virus infection in A549 cells. StA-NS2-2 raised STAT2 and MxA levels in virus infected cells independent of exogenous IFN activation (Fig. 5), suggesting that StA-NS2-2 can partially reverse the IFN signaling block imposed by rRSV-WT by inhibiting NS2 function. Finally, we sought to determine if StA-NS2-2 was capable of inhibiting RSV replication in A549 cells. As replication assays are performed over several days, we first confirmed that StA-NS2-2 retained activity for up to six days in growth media (Appendix Fig. B.2). In the first instance, we monitored virus replication via GFP reporter gene expression from the rRSV-GFP genome (Fig. 6A) and demonstrated that StA-NS2-2 modestly inhibited rRSV-GFP replication. In contrast, PIV5mCherry replication was not affected by StA-NS2-2 (Fig. 6B), consistent with our data demonstrating the compound does not reverse the IFN signaling block imposed by PIV5 V (Fig. 4B). StA-NS2-2 did not affect cell viability (Fig. 6C). Finally, to confirm the antiviral effect of StA-NS2-2 we examined infectious titer produced from compound-treated A549 cells (Fig. 6D) and IFN-deficient Vero cells (Fig. 6E). The RSV polymerase inhibitor ASL-8112 (Clarke et al., 2015, Wang et al., 2015) potently inhibited RSV in both cell types. As expected, StA-NS2-2 exhibited modest antiviral activity in A549 cells but had no significant effect in Vero cells, suggesting that StA-NS2-2 is acting solely on the IFN antagonistic function of NS2.

In summary, we have identified compounds that specifically inhibit RSV NS2 IFN antagonist function. As these compounds only partially restore IFN signaling and exhibit relatively modest antiviral activity, a medicinal chemistry campaign is now required to optimize potency. More potent compounds will enable further exploration of NS2's potential as a therapeutic target and facilitate mechanism of action studies to determine if active compounds directly bind NS2 or currently unknown host cell factors involved in NS2-mediated STAT2 degradation. Indeed, active compounds represent potentially useful chemical tools to help elucidate the unresolved molecular mechanism by which NS2 mediates STAT2 degradation. Our approach targeting RSV NS2 is novel and, to the best of our knowledge, this is the first report of compounds that inhibit RSV NS2. The successful HTS campaign targeting NS2 encouraged us to expand our study to target the unrelated CMV IFN antagonist IE1.

### 3.4 A549/pr(ISRE).GFP-derived cell-line expressing inducible CMV IE1

**Fig. 7:**
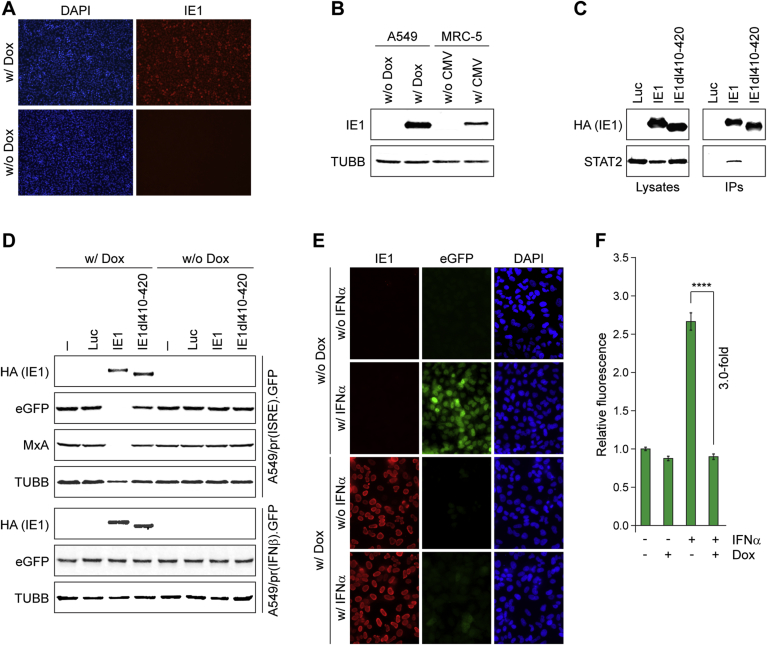
**Characterization of the A549/pr(ISRE).GFP-IE1 reporter cell-line.** (**A**) Immunofluorescence analysis of IE1 expression. A549/pr(ISRE).GFP-IE1 cells were treated with doxycycline for 24 h (w/Dox) or left untreated (w/o Dox). Paraformaldehyde-fixed samples were examined for IE1 expression using mouse anti-IE1 and Alexa Fluor 594 goat anti-mouse antibodies. Staining with 4′,6-diamidino-2-phenylindole (DAPI) was performed to visualize nuclei. (**B**) Western blot analysis of IE1 expression. A549/pr(ISRE).GFP-IE1 cells (A549) were treated with doxycycline for 24 h (w/Dox) or left untreated (w/o Dox), and MRC-5 fibroblasts were infected with gTBwt (MOI = 1 PFU/cell) for 24 h (w/CMV) or left uninfected (w/o CMV). Whole cell extracts were prepared, and IE1 and β-tubulin (TUBB) steady-state protein levels were analyzed by SDS-PAGE/Western blot. (**C**) Complex formation between IE1 and STAT2. A549/pr(ISRE).GFP cells with inducible expression of Luc, HA-tagged IE1 or HA-tagged IE1dl410-420 were treated with doxycycline for 48 h. Whole cell extracts were prepared and used for immunoprecipitations with anti-HA-agarose. Samples of lysates and immunoprecipitates (IPs) were analyzed by SDS-PAGE/Western blot for IE1 (HA antibody) and STAT2. (**D**) Western blot analysis of the effects IE1 exerts on IFN signaling and IFN induction. A549/pr(ISRE).GFP and A549/pr(IFNβ).GFP cells without (−) or with inducible expression of Luc, HA-tagged IE1 or HA-tagged IE1dl410-420 were seeded in the presence (w/Dox) or absence (w/o Dox) of doxycycline and treated with IFNα (A549/pr(ISRE).GFP cell-lines) or UV-inactivated, pp65-deficient CMV (A549/pr(IFNβ).GFP cell-lines) for 48 h. Whole cell extracts were prepared and IE1 (HA antibody), eGFP, β-tubulin (TUBB) and MxA protein levels were analyzed by SDS-PAGE/Western blot. (**E**) Immunofluorescence analysis of the effects IE1 exerts on IFN signaling. A549/pr(ISRE).GFP-IE1 cells were seeded in the presence (w/Dox) or absence (w/o Dox) of doxycycline and treated with IFNα (w/IFNα) or solvent (w/o IFNα) for 24 h. Paraformaldehyde-fixed samples were simultaneously reacted with a rat monoclonal antibody to HA-tagged IE1 and a mouse monoclonal antibody to eGFP, followed by incubation with a rat-specific Alexa Fluor 594 conjugate and a mouse-specific Alexa Fluor 488 conjugate. Host cell nuclei were visualized by DAPI staining. (**F**) Effect of IE1 expression on eGFP fluorescence intensity upon activation of the IFN signaling pathway. A549/pr(ISRE).GFP-IE1 cells were seeded in the presence or absence of doxycycline and treated with IFNα or solvent for 48 h. Fluorescence intensity was measured using an Infinite-M200-Pro microplate reader. Means and SDs of triplicate samples are shown in comparison to untreated cells (set to 1). Statistical significance was assessed using a two-tailed, unpaired/equal variance T-test; ****, p < 0.0001.

IE1 is arguably the main IFN antagonist of human CMV (Paulus et al., 2006) and an unexploited drug target. The continued presence of IE1 is incompatible with genomic integrity and normal cell proliferation (Castillo et al., 2005, Shen et al., 1997). Therefore, a doxycycline-dependent inducible IE1 expression system was used to generate an A549/pr(ISRE).GFP-IE1 derivative. IE1 expression in the presence or absence of doxycycline was validated via immunofluorescence microscopy (Fig. 7A) and Western blotting (Fig. 7B). In the absence of doxycycline, IE1 was undetectable. However, (nuclear) IE1 was readily detected in the presence of the inducer (Fig. 7A and B). Co-immunoprecipitation analysis confirmed that IE1 and STAT2 physically interact in A549/pr(ISRE).GFP-IE1 cells (Fig. 7C). In contrast, no complex formation was observed in an A549/pr(ISRE).GFP-derived cell-line where a mutant IE1 (IE1dl410-420) lacking the STAT binding motif (Harwardt et al., 2016) was expressed at levels comparable to the WT protein (Fig. 7C). As expected, IFNα treatment triggered the accumulation of eGFP in uninduced A549/pr(ISRE).GFP cells (Fig. 7D and E). Expression of WT IE1, but not IE1dl410-420 or Luc, completely blocked IFNα-induced eGFP accumulation (Fig. 7D) and fluorescence (Fig. 7E and F). To confirm specificity for the ISRE containing promoter, we generated A549/pr(IFNβ).GFP cell-line derivatives expressing inducible WT and mutant IE1. IE1 did not block eGFP accumulation induced by UV-inactivated CMV in these cells (Fig. 7D), confirming that the viral protein does not affect the type I IFN response upstream of JAK-STAT signaling (Paulus et al., 2006). These results indicate that the A549/pr(ISRE).GFP-IE1 cell-line is suitable for an HTS campaign to target CMV IE1.

### 3.5 HTS for inhibitors of CMV IE1

**Fig. 8:**
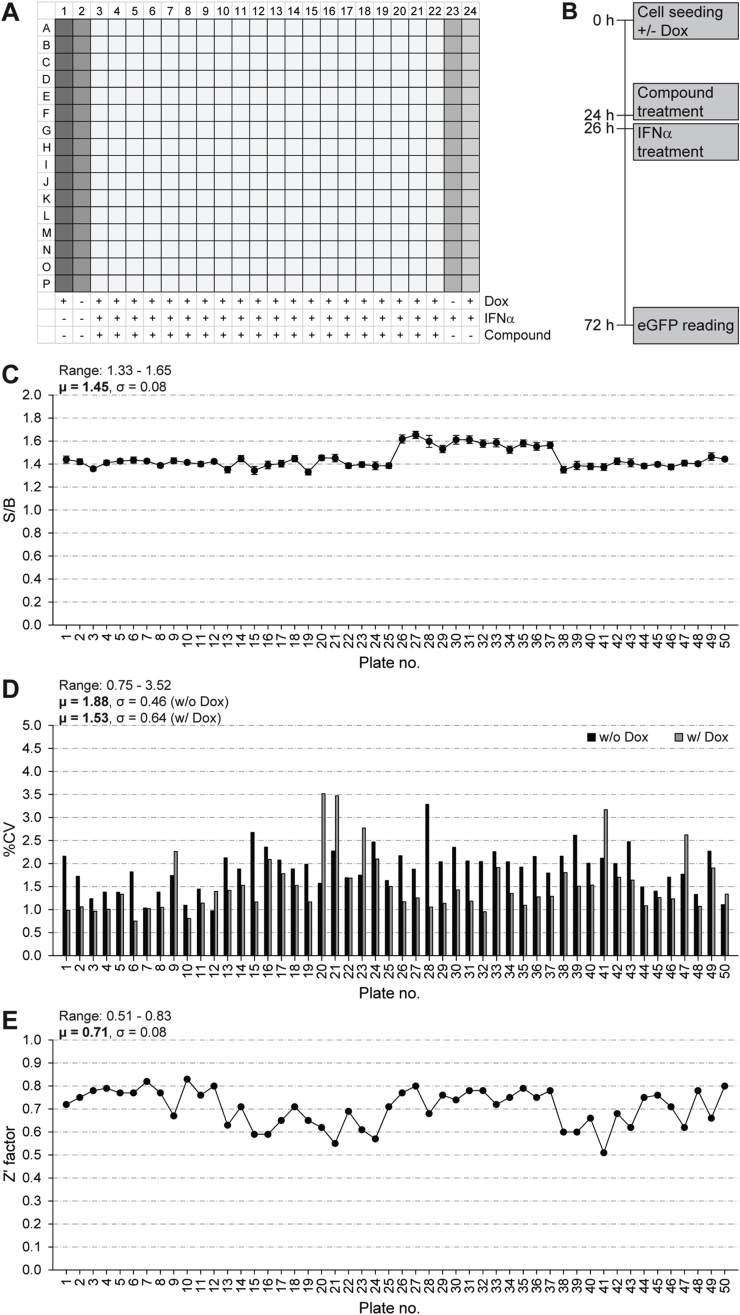
**Single-point high-throughput screen to identify compounds that inhibit the IFN signaling antagonist function of CMV IE1.** The plate layout illustrating positive and negative controls (**A**), the timing of treatments (**B**), signal-to-background (S/B) ratios of doxycycline-induced IFN-treated to uninduced IFN-treated cells (**C**), coefficients of variation (%CV) of uninduced (w/o Dox) and induced (w/Dox) cells (**D**) and Z′ Factor values (**E**) for the 50,384-well assay plates of the primary screen are shown.

**Fig. 9:**
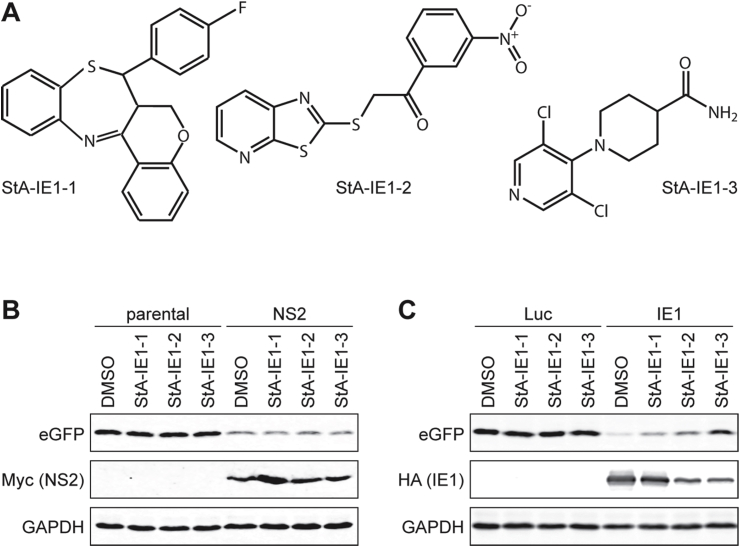
**Validation of repurchased StA-IE1 hit compounds in the A549/pr(ISRE).GFP reporter cell-lines.** (**A**) Chemical structures of hit compounds StA-IE1-1, StA-IE1-2, and StA-IE1-3. (**B**) A549/pr(ISRE).GFP reporter (parental) and A549/pr(ISRE).GFP-NS2 cells as well as (**C**) doxycycline-induced A549/pr(ISRE).GFP-Luc and A549/pr(ISRE).GFP-IE1 cells were treated with DMSO or 20 μM StA-IE1-1, StA-IE1-2 or StA-IE1-3 for 2 h before the addition of 2000 U/ml IFNα. Cells were harvested 48 h later and whole cell extracts were prepared and analyzed by SDS-PAGE/Western blot for eGFP, GAPDH, and NS2 (**B**) or eGFP, GAPDH, and IE1 (**C**).

A single-point primary HTS against the A549/pr(ISRE).GFP-IE1 cell-line was performed using the StA-HTS-Library. The 384-well plate layout with positive and negative controls and the timing of treatments are shown in Fig. 8A and B, respectively. We confirmed that the A549/pr(ISRE).GFP-IE1 cell-line is suitable for HTS via S/B ratio (Fig. 8C), CV (Fig. 8D) and Z′ Factor (Fig. 8E) analysis. Our predetermined QC criteria were achieved, with the exception of S/B ratio, which was low (μ = 1.45; QC > 2). While a high S/B ratio is preferable, the importance of this parameter is overridden by the excellent Z′ factor score (μ = 0.71; QC > 0.5), which better indicates assay quality as it takes into account both the assay signal window (S/B ratio) and data variability (Zhang et al., 1999). To be considered a hit, a compound had to restore eGFP fluorescence intensity to ≥3 SDs above the test well average of the plate. This resulted in 51 hits and a primary screen hit rate of 0.32%. A total of 33 hits were excluded from further analyses, because they were identified in other screens involving A549/pr(ISRE).GFP reporter cells. The remaining 18 compounds were cherry-picked from the StA-HTS-Library. Fifteen compounds were eliminated due to auto-fluorescence or lack of activity upon re-testing. The remaining three compounds (StA-IE1-1, StA-IE1-2 and StA-IE1-3) were repurchased (Fig. 9A).

The StA-IE1 compounds had no effect on eGFP protein levels in the parental ISRE reporter cell line and in reporter cells constitutively expressing RSV NS2 (Fig. 9B). Equally, StA-IE1-1, StA-IE1-2 and StA-IE1-3 did not affect eGFP levels in doxycycline-induced Luc control cells, but the StA-IE1 compounds restored eGFP expression in IFNα-treated A549/pr(ISRE).GFP-IE1 cells with varying efficiencies (Fig. 9C). Surprisingly, StA-IE1-2 and StA-IE1-3, which shared no obvious structural similarities (Fig. 9A), markedly reduced steady-state IE1 protein levels without affecting the accumulation of a cellular control protein (Fig. 9C).

### 3.6 Effects of hit compounds on CMV gene expression and replication

**Fig. 10:**
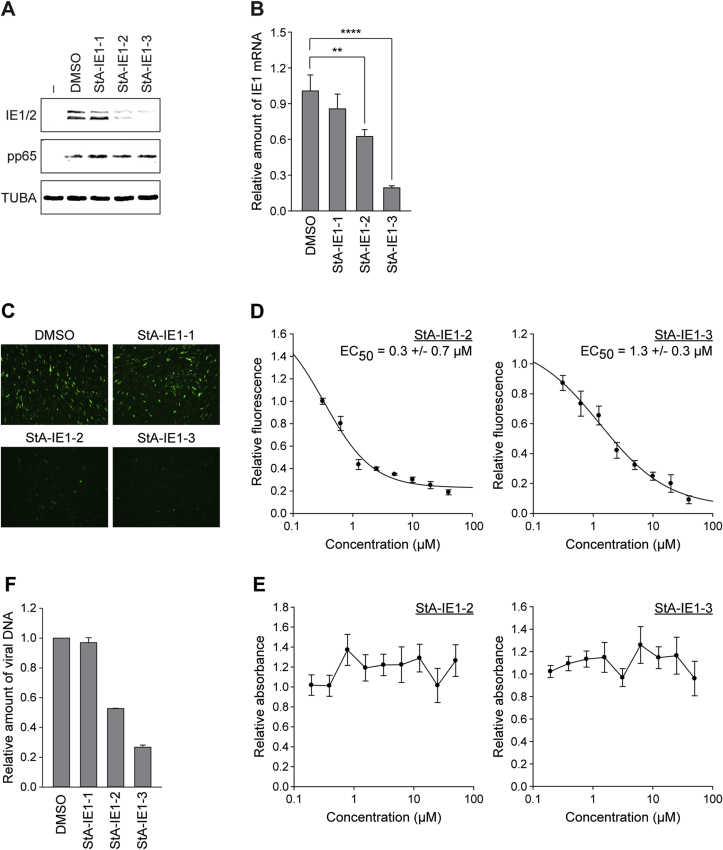
**StA-IE1-2 and StA-IE1-3 inhibit CMV major IE gene expression and CMV replication.** (**A**) MRC-5 fibroblasts were infected with gTBwt at an MOI of 1 PFU/cell in the absence (−) or presence of DMSO or 20 μM compound. Whole cell extracts were prepared at 8 h post-infection, and IE1, IE2, pp65 and α-tubulin (TUBA) protein levels were analyzed by SDS-PAGE/Western blot. (**B**) MRC-5 fibroblasts were infected with gTBwt at an MOI of 1 PFU/cell in the presence of DMSO or 20 μM compound for 8 h. Relative mRNA expression levels were determined by RT-qPCR with primers specific for IE1 exon 4. Results were normalized to β-tubulin and mean values with SDs from two biological and two technical replicates are shown in comparison to DMSO (set to 1). Statistical significance was assessed using a two-tailed, unpaired/equal variance T-test; **, p < 0.01; ****, p < 0.0001. (**C**) MRC-5 fibroblasts were infected with gTBwt at an MOI of 0.5 PFU/cell in the presence of DMSO or 20 μM compound. eGFP expression from the viral genome was assessed at 48 h post-infection by fluorescence microscopy. (**D**) MRC-5 fibroblasts were infected with gTBwt at an MOI of 0.5 PFU/cell in the presence of two-fold serial dilutions (40 μM-312.5 nM) of compound StA-IE1-2 (left) or StA-IE1-3 (right). At 72 h post-infection, cells were fixed with formaldehyde and eGFP expression from the viral genome was measured using an Infinite-M200-Pro microplate reader. Means and SDs of triplicate samples are shown in comparison to DMSO-treated cells (set to 1). (**E**) MRC-5 fibroblasts were cultivated in the presence of two-fold serial dilutions (50 μM-195.3 nM) of compound StA-IE1-2 or StA-IE1-3 for 72 h. Cells attached to the bottom of the plate were stained with crystal violet and absorbance at 570 nm was measured. Means and SDs of triplicate samples are shown in comparison to DMSO-treated cells (set to 1). (**F**) MRC-5 fibroblasts were infected with gTBwt at an MOI of 3 PFU/cell in the presence of DMSO or 20 μM compound. Viral replication was assessed at day 4 post infection by qPCR-based relative quantification of CMV DNA from culture supernatants with oligonucleotide primers specific for the viral UL86 sequence. Data are presented as means and SDs from two biological and two technical replicates relative to DMSO-treated cells (set to 1).

We investigated whether the hit compounds affect the accumulation of viral proteins including IE1 during CMV infection. MRC-5 cells were infected with CMV and subjected to Western blotting (Fig. 10A). Both StA-IE1-2 and StA-IE1-3 reduced the levels of IE1 and IE2 without affecting the accumulation of the viral tegument protein pp65 or α-tubulin. These results largely exclude off-target effects on virus entry into cells. The third hit compound, StA-IE1-1, had little effect on any of the tested proteins. The reduction of IE1 protein levels by StA-IE1-2 and StA-IE1-3 is unlikely to be a consequence of effects at the protein level, but rather results from repressed transcription since the IE1 mRNA levels also decrease in the presence of the two compounds (Fig. 10B).

To investigate whether the hit compounds exhibit antiviral activity, eGFP expression from the CMV genome was assessed by fluorescence microscopy. In the presence of StA-IE1-2 and StA-IE1-3, but not StA-IE1-1, eGFP expression was dramatically reduced compared to control cells treated with solvent (Fig. 10C). Dose response analyses revealed that CMV replication was impaired by both StA-IE1-2 and StA-IE1-3 with an EC_50_ of approximately 0.3 or 1.3 μM, respectively (Fig. 10D). The reduction in viral replication was not due to cytotoxic effects, since neither of the two effective compounds induced significant cell death within the tested range of concentrations (Fig. 10E). Consistent with the fluorescence analysis, viral particle production from cells treated with StA-IE1-2 or StA-IE1-3 was reduced compared to cells treated with StA-IE1-1 or solvent (Fig. 10F).

**Fig. 11:**
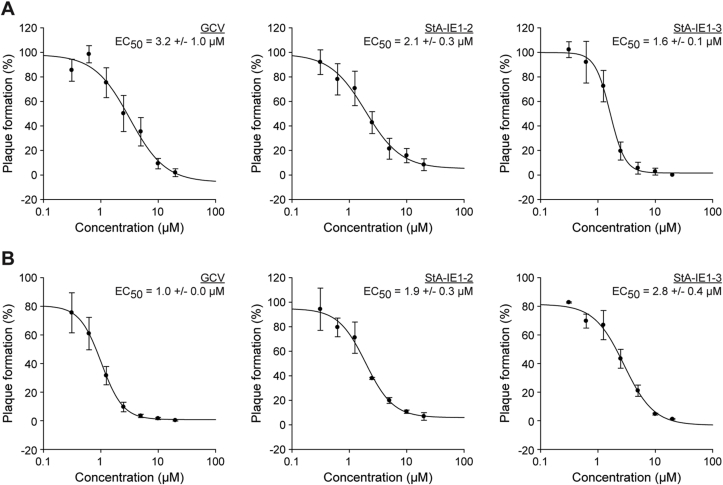
**StA-IE1-2 and StA-IE1-3 inhibit CMV plaque formation in the presence and absence of STAT2.** The plating efficiency of CMV gTBwt was determined in MRC-5 cells (**A**) and STAT2-deficient dermal fibroblasts (**B**) treated with 20 μM–312.5 nM GCV, StA-IE1-2, or StA-IE1-3. Means and SDs of triplicate measurements are shown in comparison to solvent-treated cells (set to 100%).

To further assess the antiviral effect of StA-IE1-2 and StA-IE1-3 a CMV plaque reduction assay was performed (Fig. 11). The EC_50_ of GCV, the current gold standard for the treatment of CMV diseases, was determined to be 3.2 μM, consistent with previous reports (see for example (Landry et al., 2000)). Notably, the StA-IE1 compounds were even more potent than GCV in inhibiting CMV plaque formation in MRC-5 cells with EC_50_ values of 2.1 μM and 1.6 μM for StA-IE1-2 and StA-IE1-3, respectively (Fig. 11A). The situation was slightly different in human dermal fibroblasts lacking the IE1 target STAT2. In these IFN-signaling-deficient cells the StA-IE1 compounds were outperformed by GCV which exhibited a 1.9- and 2.8-fold lower EC_50_ value compared to StA-IE1-2 and StA-IE1-3, respectively (Fig. 11B).

Taken together, our results indicate that StA-IE1-2 and StA-IE1-3 reduce viral major IE gene transcription resulting in lower IE1/IE2 protein levels and, consequently, impaired CMV replication. Since CMV IE gene products are multifunctional proteins that perform vital roles in virus replication beyond IFN antagonism, the two StA-IE1 compounds are expected to have pleiotropic antiviral effects in vivo. This view is supported by the observation that StA-IE1-2 and StA-IE1-3 are effective both in STAT2-positive and STAT2-negative cells (Fig. 11A and B).

The finding that StA-IE1-2 and StA-IE1-3 inhibit CMV replication by interfering with transcription of IE1 is unexpected given that our platform was set up to screen for inhibitors targeting IFN antagonists at the protein rather than the mRNA level. The inhibitors were presumably identified because IE1 expression is driven by major IE promoter sequences in both the lentivirus reporter used for screening, as well as the natural context of the CMV genome. Although structurally unrelated, StA-IE1-2 and StA-IE1-3 both likely act as potent inhibitors of the CMV major IE promoter, perhaps by targeting a cellular transcription factor or upstream signaling protein. Interestingly, a previous cell-based screen for inhibitors of very early events in CMV infection identified a compound (DPPC) identical to StA-IE1-3 (Fukui et al., 2008). DPPC was shown to disrupt CMV infection after viral entry but before IE gene expression, consistent with our results for StA-IE1-3. To our knowledge, StA-IE1-2 has not been previously linked to inhibition of CMV or any other virus. StA-IE1-2 and StA-IE1-3 have the potential to be further developed into novel drugs that may complement or replace existing antiviral strategies for CMV, that have traditionally targeted viral DNA replication rather than IE gene expression.

## 4 Conclusion

We have successfully developed and executed a novel modular cell-based platform that facilitates the safe and rapid screening for inhibitors of potentially any viral IFN antagonist of choice. The assay's power and flexibility stems from utilizing the same validated reporter cell-lines as a platform that can be used to rapidly generate a suite of cell-lines expressing different target viral IFN antagonists, but which readout the same pathway endpoint reporter gene. The optimal assay format utilizes inducible expression of the IFN antagonist in the reporter cell-line, as described here for CMV IE1. This is because it facilitates maximum/minimum set points and statistical parameters Z′ factor and S/B ratio (which require a signal window to be calculated) to be determined using a single reporter cell-line expressing a viral IFN antagonist of choice. Our assay negates the need to laboriously develop and validate a unique biochemical assay for each viral IFN antagonist and permits simultaneous HTS, offering power to control for off-target effects. Additionally, the assays simplicity and “off-the-shelf-potential” make them very applicable for rapidly responding to emerging viral infections and costly, time-consuming high bio-containment facilities are not required as only the target viral IFN antagonist is expressed without the need for replication competent virus. The fact that almost all viruses encode a viral IFN antagonist delivers the potential to screen for and develop compounds to treat a wide range of important viral diseases.

## Figures and Tables

**Fig. S1:**
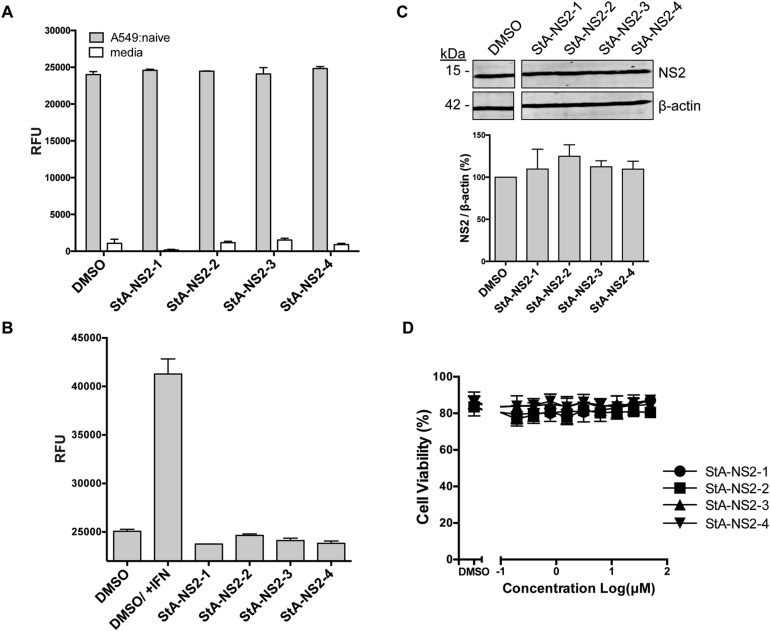


**Fig .S2:**
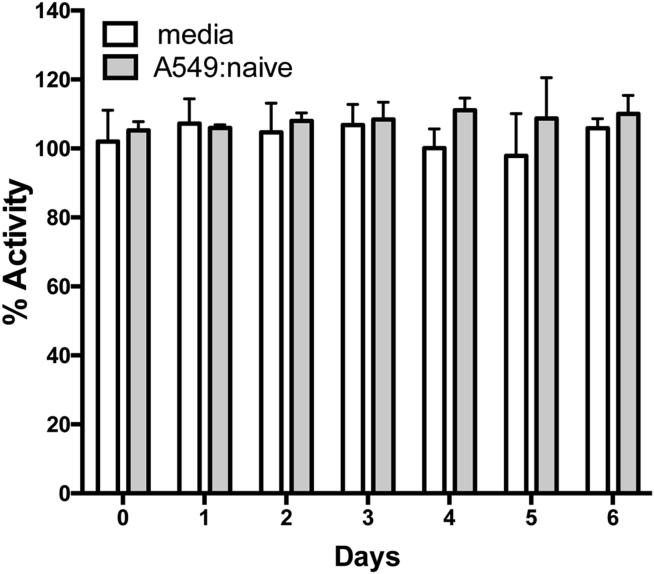


## Appendix A Supplementary dataThe following is/are the supplementary data related to this article:

mmc1 mmc1
