# Supplementary material for: Modular cell-based platform for high throughput identification of compounds that inhibit a viral interferon antagonist of choice
Source: Antiviral Res. 2018 Feb;150:79–92. doi: 10.1016/j.antiviral.2017.10.012 (PMC5800491; doi:10.1016/j.antiviral.2017.10.012)
Supplement: mmc1 [file mmc1.docx]

**Appendix A: Materials and Methods**

**Table A.1:** A549 Cell-line Derivatives

| **Name** | **Description** | **Expressed Gene Source** |
| --- | --- | --- |
| A549/pr(IFNβ).GFP | A549 derivative with eGFP gene under the control of the IFNβ promoter^1^. | Not applicable |
| A549/pr(IFNβ).GFP-Luc | A549/pr(IFNβ).GFP derivative expressing luciferase. | pLVX-TetOne-Puro-Luc vector (Clontech) |
| A549/pr(IFNβ).GFP-NS3-4A | A549/pr(IFNβ).GFP derivative expressing HCV NS3-4A with C-terminal V5 epitope tag. | GenBank accession: **AJ238799** |
| A549/pr(IFNβ).GFP-IE1 | A549/pr(IFNβ).GFP derivative expressing CMV IE1 with N-terminal HA epitope tag. | GenBank accession: **AC146851.1** |
| A549/pr(IFNβ).GFP-IE1dl410-420 | A549/pr(IFNβ).GFP derivative expressing CMV IE1 deletion mutant deficient for STAT2 binding^2^. | GenBank accession:  **AC146851.1** |
| A549/pr(ISRE).GFP | A549 derivative with eGFP gene under the control of the MxA promoter (containing IFN-stimulated response elements (ISREs))^3,4^. | Not applicable |
| A549/pr(ISRE).GFP-Luc | A549/pr(ISRE).GFP derivative expressing luciferase. | pLVX-TetOne-Puro-Luc vector (Clontech) |
| A549/pr(ISRE).GFP-NS1 | A549/pr(ISRE).GFP derivative expressing RSV NS1 with N-terminal V5 epitope tag. | GenBank accession: **AY904040.1** |
| A549/pr(ISRE).GFP-NS2 | A549/pr(ISRE).GFP derivative expressing RSV NS2 with N-terminal c-Myc epitope tag. | GenBank accession: **AY904041.1** |
| A549/pr(ISRE).GFP-NS1+NS2 | A549/pr(ISRE).GFP derivative expressing RSV NS1 and NS2 with N-terminal V5 and c-Myc epitope tags, respectively. | GenBank accession: **AY904040.1**  and **AY904041.1** |
| A549/pr(ISRE).GFP-V | A549/pr(ISRE).GFP derivative expressing PIV5 V. | GenBank accession: **JQ743318.1** |
| A549/pr(ISRE).GFP-IE1 | A549/pr(ISRE).GFP derivative expressing CMV IE1 with N-terminal HA epitope tag. | GenBank accession: **AC146851.1** |
| A549/pr(ISRE).GFP-IE1dl410-420 | A549/pr(ISRE).GFP derivative expressing CMV IE1 deletion mutant deficient for STAT2 binding^2^. | GenBank accession:  **AC146851.1** |

^1^(Chen et al., 2010), ^2^(Harwardt et al., 2016), ^3^(Stewart et al., 2014), ^4^(Gage et al., 2016), Abbreviations: GFP = Green Fluorescent Protein, HCV = Hepatitis C Virus, CMV = Cytomegalovirus, RSV = Respiratory Syncytial Virus, PIV5 = Parainfluenza Virus Type 5.

**Table A.2:** Primary Antibodies

| **Antibody** | **Manufacturer** |
| --- | --- |
| mouse anti-α-tubulin mAb | Li-Cor |
| mouse anti-GFP mAb | Roche |
| mouse anti-V5 mAb (336/SV5-PK1) | AbD Serotech |
| mouse anti-myc mAb (4A6) | Millipore |
| mouse anti-IE1/IE2 mAb 810R | Millipore |
| mouse anti-IE1 mAb 1B12 | T. Shenk, Princeton University, USA^1^ |
| mouse anti-pp65 mAb 8F5 | T. Shenk, Princeton University, USA^2^ |
| rabbit anti-β-tubulin polyclonal Ab | Li-Cor |
| rabbit anti-GAPDH polyclonal Ab | Cusabio |
| rabbit anti-Mx1/2/3 Ab (H285) | Santa Cruz Biotechnology |
| rabbit anti-STAT2 Ab (C-20) | Santa Cruz Biotechnology |
| rat anti-HA mAb 3F10 | Roche |
| goat anti-actin Ab | Abcam |
| goat anti-RSV polyclonal Ab | Abcam |

^1^(Zhu et al., 1995), ^2^(Nowak et al., 1984).

**Appendix B. Results**

**Figure B.1. Elimination of potential off-target effects associated with StA-NS2-1, -2, -3 and -4.** (**A**) Effect of compounds on fluorescence (RFU) of cell-free DMEM and A549 cells after 48 hours incubation in the presence of test compound (10 μM) or the equivalent volume of DMSO. Data shown represents mean values (n=3 replicates; error bars = SD). (**B**) Effect of compounds on eGFP expression in the A549/pr(ISRE).GFP reporter cell-line after 48 hours incubation in the presence of test compound (10 μM) or the equivalent volume of DMSO. Data shown represents mean values (n=3 replicates; error bars = SD). (**C**) Effect of compounds on NS2 and β-actin in the A549/pr(ISRE).GFP-NS2 reporter cell-line after 48 hours incubation in the presence of test compound (10 μM) or the equivalent volume of DMSO. Cells were lysed, subjected to SDS-PAGE/Western blot, followed by detection with anti-myc or anti-actin antibody and IRDye800-conjugated secondary antibody. Bands were visualized using an Odyssey near-infrared scanner, followed by quantification of % NS2 relative to β-actin. Data shown represents three independent experiments; error bars = SD. (**D**) Effect of compounds on A549 cell viability via an AlamarBlue assay after 48 hours incubation in the presence of test compound (two-fold serial dilution 50-0.1 μM) or the equivalent volume of DMSO. Data shown represents mean values (n=6 replicates; error bars = SD).

**Figure B.2. Stability of StA-NS2-2 activity.** Cell-free DMEM or A549 cells were treated with compound (10 μM) and incubated for six days. Each day supernatant was collected and stored at -20°C. Upon completion of the time-course, stored supernatant or freshly prepared media containing StA-NS2-2 was added to A549/pr(ISRE).GFP-NS2 reporter cells and the IFN signaling pathway activated with IFNα. Ability of compound within time-point supernatant samples to restore eGFP expression was determined by calculating % activity, relative to freshly prepared StA-NS2-2 media that was set at 100% activity. Cell viability was monitored by crystal violet staining (A_570_ and eGFP expression measured as RFU and subsequently normalized to cell viability values. Data shown represents mean values (n=3 replicates; error bars = SD).

**Appendix C. References**

Chen, S., Short, J.A., Young, D.F., Killip, M.J., Schneider, M., Goodbourn, S., Randall, R.E., 2010. Heterocellular induction of interferon by negative-sense RNA viruses. Virology 407, 247-255.

Gage, Z.O., Vasou, A., Gray, D.W., Randall, R.E., Adamson, C.S., 2016. Identification of Novel Inhibitors of the Type I Interferon Induction Pathway Using Cell-Based High-Throughput Screening. J Biomol Screen 21, 978-988.

Harwardt, T., Lukas, S., Zenger, M., Reitberger, T., Danzer, D., Ubner, T., Munday, D.C., Nevels, M., Paulus, C., 2016. Human Cytomegalovirus Immediate-Early 1 Protein Rewires Upstream STAT3 to Downstream STAT1 Signaling Switching an IL6-Type to an IFNgamma-Like Response. PLoS Pathog 12, e1005748.

Nowak, B., Sullivan, C., Sarnow, P., Thomas, R., Bricout, F., Nicolas, J.C., Fleckenstein, B., Levine, A.J., 1984. Characterization of monoclonal antibodies and polyclonal immune sera directed against human cytomegalovirus virion proteins. Virology 132, 325-338.

Stewart, C.E., Randall, R.E., Adamson, C.S., 2014. Inhibitors of the interferon response enhance virus replication in vitro. PLoS One 9, e112014.

Zhu, H., Shen, Y., Shenk, T., 1995. Human cytomegalovirus IE1 and IE2 proteins block apoptosis. J Virol 69, 7960-7970.
